# The chromosome-level rambutan genome reveals a significant role of segmental duplication in the expansion of resistance genes

**DOI:** 10.1093/hr/uhac014

**Published:** 2022-04-11

**Authors:** Jinfang Zheng, Lyndel W Meinhardt, Ricardo Goenaga, Tracie Matsumoto, Dapeng Zhang, Yanbin Yin

**Affiliations:** 1 Nebraska Food for Health Center, Department of Food Science and Technology, University of Nebraska, Lincoln, NE 68588, USA; 2 USDA-ARS, Sustainable Perennial Crops Laboratory, Beltsville, MD 20705, USA; 3 USDA-ARS, Tropical Agriculture Research Station, Mayaguez, PR 00680; 4 USDA-ARS, Daniel K. Inouye Pacific Basin Agricultural Research Center, Hilo, 96720, HI, USA

Dear Editor,

Rambutan (*Nephelium lappaceum* var. *lappaceum*), a tropical fruit tree native to southeastern Asia, belongs to the family Sapindaceae. Rambutan is a popular table fruit and is also processed into preserves, juices, wines, and sorbets [1]. At present, only three Sapindaceae genomes are publicly available: *Xanthoceras sorbifolium* [2], *Dimocarpus longan* (longan) [3], and *Acer yangbiense* [4]. During the process of submitting this manuscript, the genome paper for the rambutan cultivar Baoyan7 became available online, but its genome sequence has not yet been released [5].

Here, we sequenced the rambutan cultivar R-162 (Figure S1) sampled from Puerto Rico (USDA-ARS, Tropical Agriculture Research Station, Mayaguez) in order to study the genomic expansion of plant resistance genes. The R-162 genome has a scaffold N50 of 21.65 Mb and 16 pseudo-chromosomes. The chromosome-level genome was assembled using Dovetail Genomics HiRise scaffolding software with long-range Chicago libraries and Hi-C library sequencing. Genome annotation identified 49,959 protein-coding genes, 38,075 of which were annotated by eggNOG and 33,429 of which were supported by RNA-seq data. BUSCO (Benchmarking Universal Single-Copy Orthologs) [6] analysis identified 1,573 single-copy orthologous genes, corresponding to a genome completeness of 97.5%. The genome and annotation data can be accessed at https://bcb.unl.edu/Nla/ and NCBI (BioProject ID: PRJNA766632).

For comparative genomics analysis, we collected 10 genomes (Figure 1A) from the order Sapindales, to which *N. lappaceum* belongs. These included three plants from Sapindaceae, two from Rutaceae, one from Meliaceae, one from Burseraceae, and three from Anacardiaceae. *Arabidopsis thaliana* was included as the outgroup in the phylogeny (Figure 1A).

**Figure 1:**
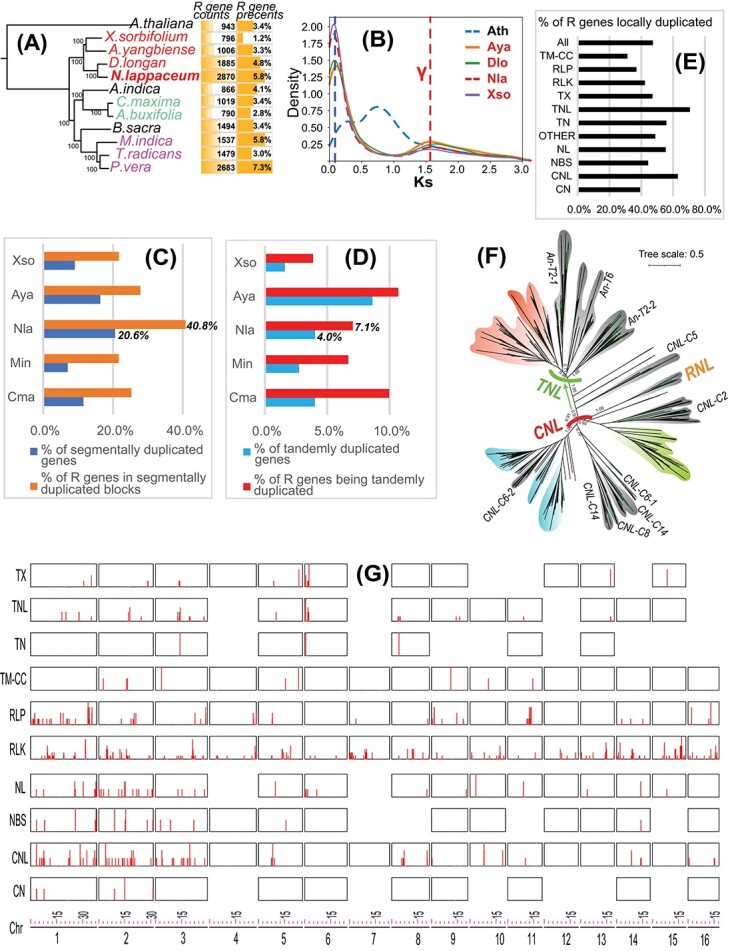
Local duplications and R genes in the genome of rambutan cultivar R-162. (**A**) A species tree was constructed using single-copy genes from 12 genomes (red: Sapindaceae, purple: Anacardiaceae, green: Rutaceae, black: others). The R gene counts and percentages are shown beside the species. The three letter species names are Abu: *Atalantia buxifolia*, Ain: *Azadirachta indica*, Ath: *Arabidopsis thaliana*, Aya: *Acer yangbiense*, Bsa: *Boswellia sacra*, Cma: *Citrus maxima*, Dlo: *Dimocarpus longan*, Min: *Mangifera indica*, Nla: *Nephelium lappaceum*, Pve: *Pistacia vera*, Tra: *Toxicodendron radicans*, and Xso: *Xanthoceras sorbifolium*. (**B**) Ks distributions of paralogous gene pairs identified by RBH were plotted for the four Sapindaceae genomes and Ath. (**C**) Five Sapindales genomes with chromosome-level assemblies were analyzed to retrieve intra-chromosomal syntenic blocks identified by WGDI (see main text). The percentages of segmentally duplicated genes are calculated as the number of genes forming homologous gene pairs in intra-chromosomal syntenic blocks with Ks < 0.2 divided by the total number of genes in a genome. R genes are plant resistance genes identified by RGAugury (see main text). The percentages of R genes in segmentally duplicated blocks are calculated as the number of R genes in segmentally duplicated blocks divided by the total number of R genes in the genome. (**D**) is similar to (C) but for tandemly duplicated genes (co-localized homologous genes with Ks < 0.2 and a gene distance <= 10). (**E**) Percentages of the 11 R gene families that are locally duplicated. The full names of the 11 R gene families are given in the main text. (**F**) Phylogeny of NBS-containing R genes from Ath and Nla. The clade labels are based on the Ath genes studied in (Shao et al., 2016) [8]. Clades that contain Ath genes are shown with a gray background, and clades only found in Nla are shown in a colored background. (**G**) Chromosomal location plot of R gene families. Each row shows the graphs with the sum count of R genes in a window size of 100 kb with stride = 100 kb.

Whole genome duplications (WGDs) were analyzed based on paralogous gene synonymous substitution (Ks) analysis using a reciprocal best hit (RBH) approach. Unlike *A. thaliana*, which is known to have undergone two additional WGDs after the γ event shared by all dicot plants (Figure 1B, red vertical line), there were no additional recent WGD events in Sapindaceae. However, all four Sapindaceae genomes have a much higher peak at Ks value <0.1 (Figure 1B, blue vertical line), which represents recent local duplications.

Local duplications are therefore more interesting in Sapindales genomes. Unlike WGDs, local duplications often occur within the same chromosomes. We have identified two types of locally duplicated intra-chromosomal segments in five genomes with chromosome-level assemblies (Nla, Aya, Xso, Cma, and Min). The two types of local duplications are: (i) colinear gene syntenic blocks identified by WGDI [7] (Figure S2), which represent segmentally duplicated segments with Ks < 0.2, and (ii) co-localized homologous genes with Ks < 0.2 and a gene distance <= 10 (the two homologous genes are less than 10 genes apart on the chromosome), which represent tandemly duplicated segments.

In total, 10 286 (20.6% of 49 959, Figure 1C) Nla genes were found to be homologous gene pairs in 13 511 intra-chromosomal syntenic blocks with Ks < 0.2, indicating that they were derived from segmental duplications. By contrast, only 2010 (4% of 49 959, Figure 1D) tandemly duplicated genes (Ks < 0.2 and gene distance <= 10) were identified in Nla. Moreover, 1516 (75.4%) of this set of tandemly duplicated genes were also found in the 10 286 segmentally duplicated genes that reside in syntenic blocks. The other four Sapindales genomes (Aya, Xso, Cma, and Min) had lower percentages (Figure 1C) of segmental gene duplications than Nla. All of them had higher percentages of segmental gene duplications (Figure 1C, blue bars) than tandem gene duplications (Figure 1D, cyan bars). Clearly, segmental gene duplications have had a larger impact on the evolution of Sapindales genomes than tandem gene duplications.

We performed a systematic search for plant resistance genes (R genes) in the 11 Sapindales genomes using RGAugury [9]. Based on the classifications defined in RGAugury, these R genes included members of 11 families: CN (CC-NBS, coiled-coil nucleotide-binding site), CNL (CC-NBS-LRR, CC-NBS leucine-rich repeat), NBS (nucleotide-binding site), NL (NBS-LRR), OTHER (others), TN (TIR-NBS, Toll/Interleukin-1 receptor NBS), TNL (TIR-NBS-LRR), TX (TIR-unknown domain), RLK (receptor-like kinase), RLP (receptor-like protein), and TM-CC (transmembrane coiled-coil). However, it should be noted that the NBS-containing families (CN, CNL, NBS, NL, TN, TNL, and some OTHERs) are more often grouped into three larger classes (TNL, CNL, and RNL) based on their other domains [8]. Here, we have adopted the RGAugury classification.

Interestingly, 40.8% (Figure 1C, orange bars) of the R genes in Nla are located in segmentally duplicated syntenic blocks, and 7.1% (Figure 1D, red bars) of the R genes are tandemly duplicated. Altogether, 47.3% of the R genes are locally duplicated (Figure 1E). These results indicate that local duplications have played a very significant role in the expansion of plant resistance gene families in Nla and that segmental gene duplications have been more important than tandem duplications. Between 20% and 28% of the R genes in other Sapindales genomes (Figure 1C) are located in segmentally duplicated syntenic blocks, much lower than the 40.8% in Nla. This finding suggests that segmental gene duplications have had a larger impact on the evolution of the Nla genome and have contributed more to the expansion of plant resistance genes in Nla than in other Sapindales genomes. It should be noted that the rate of duplication is likely to have been underestimated, given that more ancient duplicated genes are often lost. As the duplicated syntenic blocks in Sapindales genomes have very small Ks values (Figure S2 and Figure 1B), this phenomenon may be less of an issue in the present study.

Among the 11 RGAugury gene families, TNL and CNL contain the highest percentages (70.6% for TNL and 63.0% for CNL) of locally duplicated genes (Figure 1E) in the Nla genome. There are 197 TNL genes and 397 CNL genes, which are also the most abundant among the 12 genomes. The phylogeny of NBS-containing RGAugury genes (Figure 1F) shows that, compared with Ath, Nla contains a few unique and significantly expanded CNL and TNL subfamilies. Locating all R gene families on the chromosomes revealed an uneven distribution (or clustering) of R genes in the genome (Figure 1G). Chromosomes 1, 2, and 3 contain the most R genes, especially in the NBS-containing families such as NBS, CNL, NL, and TNL, whereas RLK and RLP genes are more widely distributed in all chromosomes. Clearly, local duplication has played a major role in the lineage-specific expansion of NBS-LRR genes in Nla. Comparison of the total R gene repertoires among different Sapindales genomes revealed that Nla has the largest number (2870) of R genes and the second highest R gene percentage (5.8%) of the 12 genomes (Figure 1A), probably as a result of recent local gene duplications. These findings are consistent with and significantly expand upon what has been reported in a previous study [10] of NBS-LRR gene evolution in three Sapindaceae genomes (Aya, Dlo, and Xso).

In summary, the chromosome-level assembly of the Nla genome provides a useful reference for the study of Sapindaceae plants. The draft genome will also be a great resource for the discovery of molecular markers (e.g. SNPs) for characterizing different rambutan cultivars throughout the world. The Nla resistance genes identified in this study will help researchers to develop better strategies for improving rambutan resistance to pathogens and diseases.

## Acknowledgements

We would like to acknowledge all our lab members for helpful discussions. This work was partially completed using the Holland Computing Center of the University of Nebraska, which receives support from the Nebraska Research Initiative. This work was primarily supported by a United States Department of Agriculture (USDA)/Agricultural Research Service (ARS) award [58-8042-9-089] and partially by a National Science Foundation (NSF) CAREER award [DBI-1933521], a National Institutes of Health (NIH) R01 award [R01GM140370], and a start-up grant from UNL [2019-YIN] to Y.Y. Mention of trade names or commercial products in this publication is solely for the purpose of providing specific information and does not imply recommendation or endorsement by the U.S. Department of Agriculture. The USDA is an equal opportunity provider and employer.

## Author contributions

YY, DZ, LWM, RG, and TM conceived and designed the project. DZ, LWM, and RG collected the plant materials and generated the sequencing data. JZ performed all the data analysis under the supervision of YY. JZ and YY drafted the manuscript. All authors contributed to and approved the final manuscript.

## Data availability

The raw DNA sequencing reads and the assembled genome of *N. lappaceum* cultivar R-162 have been submitted to NCBI. The BioProject ID is PRJNA766632, the BioSample ID is SAMN21855570, and the Whole Genome Shotgun accession number will be available upon publication of the paper. The genome and annotation data can also be accessed at https://bcb.unl.edu/Nla/.

## Conflict of interest statement

No conflicts of interest are declared.

## Supplementary data

Supplementary data are available at *Horticulture Research * online.

## Supplementary Material

Web_Material_uhac014
