# Supplementary material for: The chromosome-level rambutan genome reveals a significant role of segmental duplication in the expansion of resistance genes
Source: Hortic Res. 2022 Apr 11;9:uhac014. doi: 10.1093/hr/uhac014 (PMC9002659; doi:10.1093/hr/uhac014)
Supplement: Web_Material_uhac014 [file web_material_uhac014.zip › SI.docx]

**The chromosome-level rambutan genome reveals a significant role of segmental duplication in the expansion of resistance genes**

*Jinfang Zheng^1^, Lyndel W. Meinhardt^2^, Ricardo Goenaga^3^, Tracie Matsumoto^4^, Dapeng Zhang^2,*^, Yanbin Yin^1,*^*

*^1^Nebraska Food for Health Center, Department of Food Science and Technology, University of Nebraska, Lincoln, NE 68588, USA*

*^2^USDA-ARS, Sustainable Perennial Crops Laboratory, Beltsville, MD 20705, USA*

*^3^USDA-ARS^,^ Tropical Agriculture Research Station, Mayaguez, PR 00680*

*^4^USDA-ARS, Daniel K. Inouye Pacific Basin Agricultural Research Center, Hilo, 96720, HI, USA*

**Supplementary Methods**

**Plant material, 10x library prep and sequencing**

Three leaf samples of *N. lappaceum* cultivar “R-162” (**Figure S1**) were collected from the USDA-ARS Tropical Agriculture Research Station in Mayaquez, Puerto Rico. Whole genome sequencing libraries were prepared using Chromium Genome Library & Gel Bead Kit v.2 (10X Genomics, cat. 120258) and sequenced on a NovaSeq6000 sequencer (Illumina, San Diego, CA) with paired-end 150 bp reads.

**Chicago library preparation and sequencing**

Two Chicago libraries were prepared as described previously (Putnam et al., 2016). The libraries were sequenced on an Illumina HiSeq X. The number and length of read pairs produced for each library was: 175 million, 2x150 bp for library. This Chicago library reads provided 80.06x physical coverage of the genome (1-100 kb pairs).

**Dovetail Hi-C library preparation and sequencing**

Two Dovetail Hi-C libraries were prepared in a similar manner as described previously (Putnam et al., 2016). The libraries were sequenced on an Illumina HiSeq X. The number and length of read pairs produced for each library were: 248 million, 2x150 bp for library. This Dovetail Hi-C library reads provided 48,950.25X physical coverage of the genome (10-10,000 kb pairs).

**Scaffolding the assembly with HiRise**

The input *de novo* assembly, shotgun reads, Chicago library reads, and Dovetail Hi-C library reads were used as input data for HiRise, a software pipeline designed specifically for using proximity ligation data to scaffold genome assemblies (Lieberman-Aiden et al., 2009). An iterative analysis was conducted.

**Supplementary Figures**


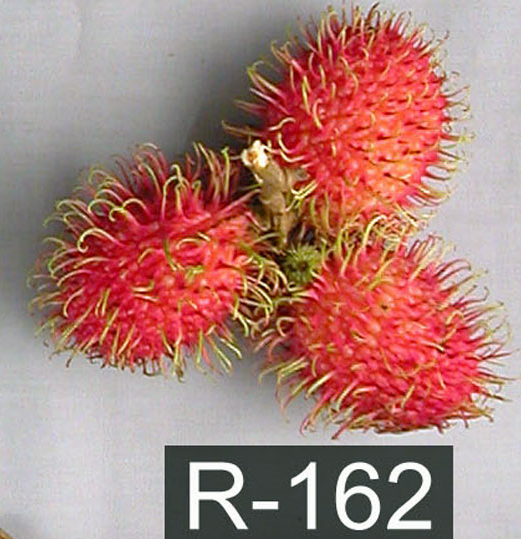


Figure S1. Fruit of cultivar “R-162”.


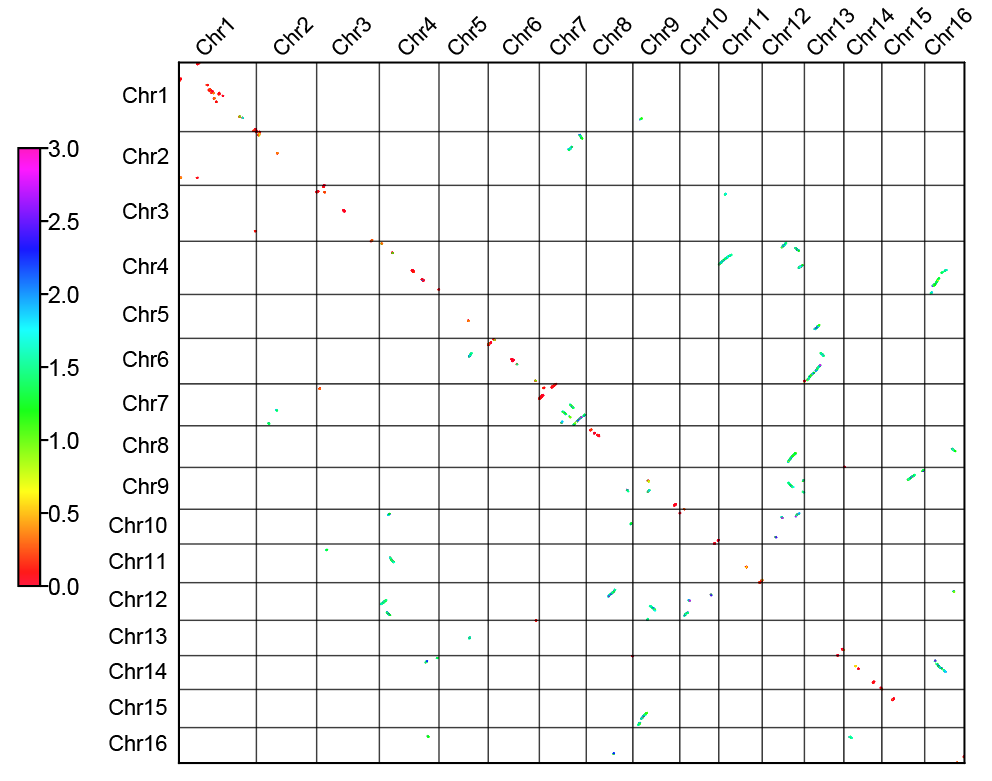


Figure S2. Dot plot of syntenic blocks in cultivar “R-162” genome.


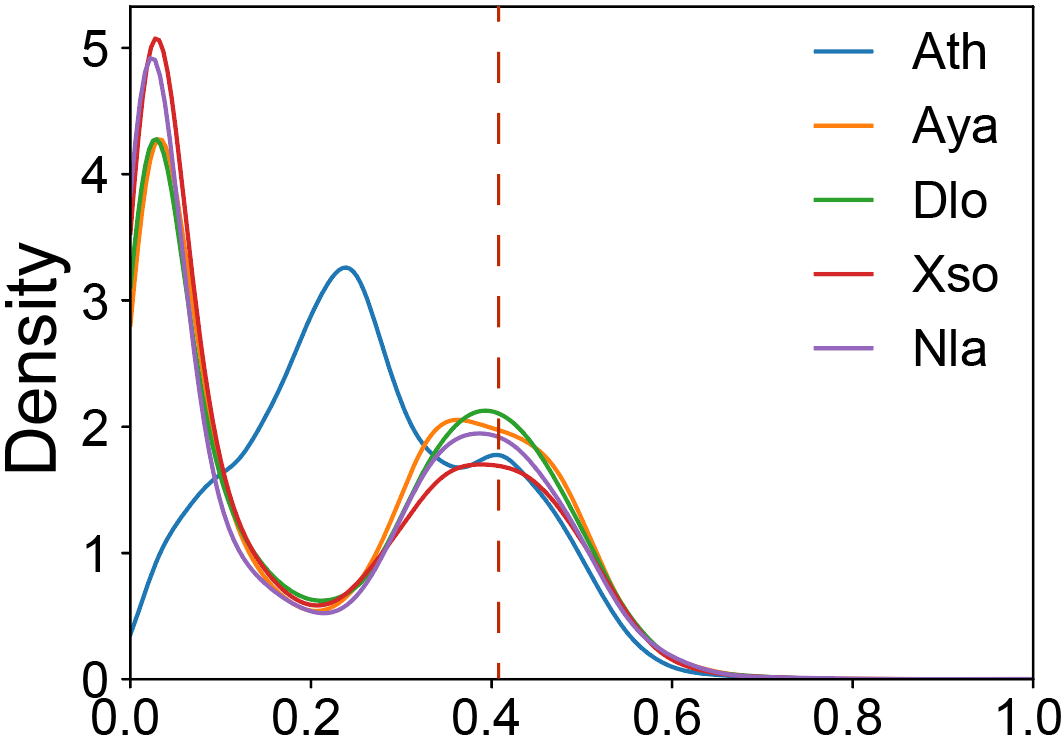


Figure S3. Distribution of 4dTv of five genomes.

**Reference**

Lieberman-Aiden, E., van Berkum, N.L., Williams, L., Imakaev, M., Ragoczy, T., Telling, A., Amit, I., Lajoie, B.R., Sabo, P.J., Dorschner, M.O.*, et al.* (2009). Comprehensive mapping of long-range interactions reveals folding principles of the human genome. Science *326*, 289-293.

Putnam, N.H., O'Connell, B.L., Stites, J.C., Rice, B.J., Blanchette, M., Calef, R., Troll, C.J., Fields, A., Hartley, P.D., Sugnet, C.W.*, et al.* (2016). Chromosome-scale shotgun assembly using an in vitro method for long-range linkage. Genome Res *26*, 342-350.
