# Supplementary material for: The chromosome-level rambutan genome reveals a significant role of segmental duplication in the expansion of resistance genes
Source: Hortic Res. 2022 Apr 11;9:uhac014. doi: 10.1093/hr/uhac014 (PMC9002659; doi:10.1093/hr/uhac014)
Supplement: Web_Material_uhac014 [file web_material_uhac014.zip › response.docx]

Dear Editor,

Thank you and your reviewers for the valuable comments on our revised "letter to editor" manuscript (**HR-2021-008.R1**). We provided in this document the point-to-point responses in green to the reviewers’ comments. The changes made in the main text are highlighted in red in the revised manuscript file. I hope that you will find our paper acceptable to be published in Horticulture Research.

Best regards,

Yanbin Yin, Ph.D.

Associate Professor

Nebraska Food for Health Center

Department of Food Science and Technology

University of Nebraska-Lincoln, USA

**Reviewer: 1**

Comments to the Author
The chromosome-level of Rambutan genome provides a valuable resource to study this tropical fruit. The MS is also written in good English. However, some more details should be provided about the genome, and some background about R genes seems not clarified, leading to the confusion of R gene classification.

**Question (Q1)**. Authors should clarify what sequencing technology they used, how many data were generated for this genome.

***Answer (A1)****: Due to the length limit on “letter to editor” type of manuscript, we had left out a lot of details. In brief, a Chromium Genome Library was used for whole genome sequencing, followed by two Chicago libraries and a Dovetail Hi-C library sequencing for scaffolding. We have now added detailed description of sequencing data in a supplementary data file.*

**Q2.** The protein-coding gene annotation detected 49,959 genes, which seems a very large number for me. Authors should provide statistic numbers, including the number of genes by homologous annotation, and the number supported by RNA-seq data.

***A2****: We added these numbers in lines 46-47.*

**Q3**. As the 49,959 genes are too many for me, and the Ks plot did show a recent peak. Although the author declared no more WGDs after γ, it still looks suspicious to me. I would like to see the intra-genomic synteny analysis.

***A3****: Here we show the dot plot of the syntenic blocks inferred by WGDI (*[*https://wgdi.readthedocs.io/en/latest/index.html*](https://wgdi.readthedocs.io/en/latest/index.html)*). The syntenic blocks are colored according to the Ks value of gene pairs in the syntenic blocks. As it is shown in below figure, most of the low Ks gene pairs are on the diagonal line (the same chromosomes), indicating local duplications instead of WGD. The green blocks not on the diagonal line indicate the γ event. We have added this figure as* ***Figure S2*** *in a supplementary data file.*

Figure S2. Dot plot of syntenic blocks in cultivar “R-162” genome.

**Q4**. The classification of R genes are confusing, the authors seem not really know about R genes. NL, CN, CNL, TN, TNL all belong to the NBS-LRR gene family. In angiosperms, NBS-LRR genes can only be phylogenetically classified into three subfamilies, CNL, TNL and RNL, although each subfamily may have truncated genes like NL, TN, CN, N, etc. (see Large-Scale Analyses of Angiosperm Nucleotide-Binding Site-Leucine-Rich Repeat Genes Reveal Three Anciently Diverged Classes with Distinct Evolutionary Patterns) I suggest the authors to reorganize the results of this analysis.

***A4****: Thank you for the comment. We have now clearly stated that NBS-LRR genes are often classified into CNL, TNL and RNL, in lines 113-116. To further study the classification of Nla NBS-LRR genes, we have built a phylogenetic tree using Nla and Ath proteins. The phylogeny is also annotated according to the suggested paper (Shao et al., 2016). Also, according to the Associate Editor, we have now included the phylogeny as* ***Figure 1F****.*

**Q5**. The different R gene number with Zhou’s results is Zhou et al. only investigated NBS-LRR gene, whereas other types, e.g. RLK, STK, were not included.

***A5****: Thank you for the comment. We have made the correction in line 145.*

**Reviewer: 2**

Comments to the Author

Journal: Horticulture research

Title: The chromosome-level rambutan genome reveals a significant role of segmental duplication in the expansion of resistance genes
Authors: Jinfang Zheng, Lyndel W. Meinhardt, Ricardo Goenaga, Tracie Matsumoto, Dapeng Zhang*, Yanbin Yin*
The work by Yin et al presents a draft genome sequence for rambutan, a member of the Sapindaceae family, which is a popular fruit for its unique traits in preserves, juices, wines and sorbets. The genome sequence was assembled by using a combination of 10X chromium sequencing and Hi-C technologies. The assembled genome is 409.27 Mb genome with a scaffold N50 value of 21.65 Mb and a total of 49,959 protein-coding genes was predicted from the genome. It is very useful for elucidating the molecular mechanisms responsible for anthocyanin biosynthesis. This study paves a road for the evolution and diversification of Sapindaceae. I recommend publish subject to a major revision. My comments are given below:

**Q1**. How much was chloroplast/mitochondria DNA sequenced? Any evidence of nuclear plastid transfer? Plastids were not even discussed. Was DNA purified from nuclei?

***A1****: We appreciate these questions. The DNA was not purified from nuclei only. The chloroplast genome has been published already (MT936934.1). Mapping raw reads to this chloroplast genome found 5.78% reads can be mapped. To find nuclear plastid gene transfer, we aligned the chloroplast genome to our assembled nuclear genome. We found that several nuclear genomic fragments are very similar (but not identical) to the chloroplast genome, indicating potential nuclear plastid transfers. Due to the word limit (total 1,200 words for “letter to editor”), this analysis is not included in this paper.*

**Q2**. Would you please confirm if rambutan has rapid genome evolution, which involves accelerated substitution rate of nucleotides, chromosomal reshuffling, fusion or break down, high activity of transposable elements, etc?

***A2****: While our manuscript was under review, the rambutan genome of a Chinese cultivar called Baoyan7 was released in GenBank (ID: GWHBECQ00000000). Comparing this Baoyan7 genome (*[*https://pubmed.ncbi.nlm.nih.gov/34519122/*](https://pubmed.ncbi.nlm.nih.gov/34519122/)*) with ours from Central America (cultivar R-162) found that the two genomes are generally well-aligned. However, some regions in Chr2, Chr3 and Chr7 are inverted (see below Figure 1). In addition, the Baoyan7 paper has compared genomes of rambutan and lychee, and revealed chromosome fission/fusion events. All these indicate a rapid genome evolution of rambutan. However, per the Editor in Chief, our manuscript has been rewritten as a short “letter to editor”, which has the word limit < 1,200 words, one figure, and 10 references. Thus, these genome evolutionary analyses are not included in this revised paper.*

*Figure 1 (for review only). Comparison of rambutan genomes of Baoyan7 (x-axis) and R-162 (y-axis).*

**Q3**. Could you please provide the 4dTv distance figure to display the WGD of these species in Figure2?
***A3****: We have included the 4dTv plot as the* ***Figure S3*** *in the supplementary data file.*

**Q4**. Would you please give more reasons to confirm that tandem gene duplications had less significant role in the evolution of Sapindales genome and the expansion of plant resistance genes than segmental gene duplications?

***A4****: Both tandem duplication and segmental duplication have played an important role in the evolution of plant resistance genes (Cannon et al., 2004). However, segmental duplication affects more genes than tandem duplication, as the latter happens more locally and on a smaller scale. However, like whole genome duplication that can affect all the genes in the genome, duplicated genes derived from more ancient segmental duplication could be lost and difficult to detect (Baumgarten et al., 2003). Therefore, it is likely that segmentally duplicated genes are often under-estimated. However, in genomes of Sapindales such as rambutan, segmental duplication occurred more recently (low Ks values in Figure 1B and Figure S2), and thus are easier to detect. We have added two sentences in lines 126-129 to explain this.*

**Q5**. Would you please verify **anthocyanin biosynthesis** genes up-regulated in red peel rambutan by experiment?

***A5****: In this short “letter to editor”, the analysis of anthocyanin biosynthesis genes is removed.*

**Associate Editor:** 
Comments to the Author:
This MS reports the genome sequence of a tropical and economic fruit rambutan. The authors have contributed a genome paper on dragon fruit previously. The most dominant finding of this MS is that the expansion of R genes.
However, I have a few comments.
**Q1**. The analyses on WGD is not enough. The authors should provide a whole genomic synteny analyses to show that no recent WGD occur.

***A1****: Please see* ***Figure S2*** *in the supplementary data file, and also our response to reviewer #1.*

**Q2**. In Figure 1a, please provide full name for these species, at least full genus name or full species name.

***A1****: Done as suggested.*

**Q3**. please provide a **fig. 1g** in which a phylogenetic tree of R genes could well show the classification of R genes in either CNL, RNL, or TNL.

***A3****: Please see* ***Figure 1F****, and also our response to reviewer #1.*

**Reference**

Baumgarten, A., Cannon, S., Spangler, R., and May, G. (2003). Genome-level evolution of resistance genes in Arabidopsis thaliana. Genetics *165*, 309-319.

Cannon, S.B., Mitra, A., Baumgarten, A., Young, N.D., and May, G. (2004). The roles of segmental and tandem gene duplication in the evolution of large gene families in Arabidopsis thaliana. BMC Plant Biol *4*, 10.
